# Guiding Device for the Patellar Cut in Total Knee Arthroplasty: Design and Validation

**DOI:** 10.3390/bioengineering5020038

**Published:** 2018-05-10

**Authors:** Erica L. Rex, Cinzia Gaudelli, Emmanuel M. Illical, John Person, Karen C. T. Arlt, Barry Wylant, Carolyn Anglin

**Affiliations:** 1 Biomedical Engineering and McCaig Institute for Bone and Joint Health, University of Calgary, Calgary, AB T2N 1N4, Canada; rexe4@hotmail.com (E.L.R.); karen.ct.arlt@gmail.com (K.C.T.A.); 2 Section of Orthopaedic Surgery, Cumming School of Medicine, University of Calgary, Calgary, AB T2N 1N4, Canada; cinzia.gaudelli@gmail.com; 3 Department of Orthopaedic Surgery, SUNY Downstate Medical Center, Brooklyn, NY 11203, USA; eillical@gmail.com; 4 Box 13 Engineering, Calgary, AB T3L 2P5, Canada; john@box13eng.com; 5 Q Industrial Design Corporation, Calgary, AB T2T 0E7, Canada; bwylant@ucalgary.ca; 6 Department of Civil Engineering, University of Calgary, Calgary, AB T2N 1N4, Canada

**Keywords:** patella, resection, device, total knee arthroplasty, validation

## Abstract

An incorrect cut of the patella (kneecap) during total knee arthroplasty, affects the thickness in different quadrants of the patella, leading to pain and poor function. Because of the disadvantages of existing devices, many surgeons choose to perform the cut freehand. Given this mistrust of existing devices, a quick, but accurate, method is needed that guides the cut, without constraining the surgeon. A novel device is described that allows the surgeon to mark a line at the desired cutting plane parallel to the front (anterior) surface using a cautery tool, remove the device, and then align the saw guide, reamer, or freehand saw with the marked line to cut the patella. The device was tested on 36 artificial patellae, custom-molded from two shapes considered easier and harder to resect accurately, and eight paired cadaveric specimens, each in comparison to the conventional saw guide technique. The mediolateral angle, superoinferior angle, difference from intended thickness, and time were comparable or better for the new device. Addressing the remaining outliers should be possible through additional design changes. Use of this guidance device has the potential to improve patellar resection accuracy, as well as provide training to residents and a double-check and feedback tool for expert surgeons.

## 1. Introduction

In total knee arthroplasty (TKA), worn and damaged surfaces of the knee are resected and replaced with artificial components. An incorrect cut of the patella, whether tilted, too thick, or too thin, occurs in at least 10% of cases, even amongst expert surgeons, leading to numerous clinical complications, in particular, a reduced range of motion, anterior knee pain, and patellofemoral impingement [1,2,3,4,5,6,7]. When rising from a low chair, only 15% of patients with a symmetric patellar cut experienced anterior knee pain compared with 44% of those with an asymmetric profile [4].

A symmetric cut is considered to be parallel to the front (anterior) surface of the patella [3,4,8]; however, existing devices either have no connection with the anterior surface or the contact points can cause the device to tilt because of high or low points on the surface [8,9,10]. A patellar computer-assisted surgery system addressed many of these factors, but the screw attachment of the bone reference frame was considered invasive; a better system to define the anterior surface was desired; and the greater simplicity of a mechanical interface was attractive [10,11].

Three main techniques are currently used to resect the patella: freehand with a saw, using a saw guide, and using a reamer [10]. None of these techniques has gained widespread acceptance because each has its own disadvantages. The freehand technique relies entirely on the experience of the surgeon. Achieving good accuracy is difficult because of the lack of distinctive landmarks, the small size of the patella, and the hard bone. The saw guide also depends on the subjective experience of the surgeon; it can be difficult to clamp securely around the patella; and it may be difficult to position at the correct height without further dissection of the soft tissues. The reamer is the easiest to apply, but can be inadvertently tilted, without the user realizing it, and can give the incorrect depth due to variability in the depth of the spikes [8,10].

On the basis of feedback from surgeons and residents, we developed a device with the objectives of guiding the cut rather than constraining it, so that surgeons can continue to use their judgment; be fast and simple to use; have minimal impact on the surgeon’s current surgical procedure; provide a training tool for residents and a double-check and feedback tool for expert surgeons; and be able to be used either with everting the patella (as in conventional surgery) or without everting the patella (as in minimally-invasive surgery). 

The purpose of this study was to evaluate the device using artificial bone models and cadaveric specimens, to determine the accuracy, the ease of use, and potential design improvements. Accuracy was judged by the mediolateral (ML) resection angle, superoinferior (SI) resection angle, and the difference from the intended thickness. The hypothesis was that the new device is more accurate than existing techniques, while taking comparable or less time.

## 2. Materials and Methods

### 2.1. Device Design

**Figure 1:**
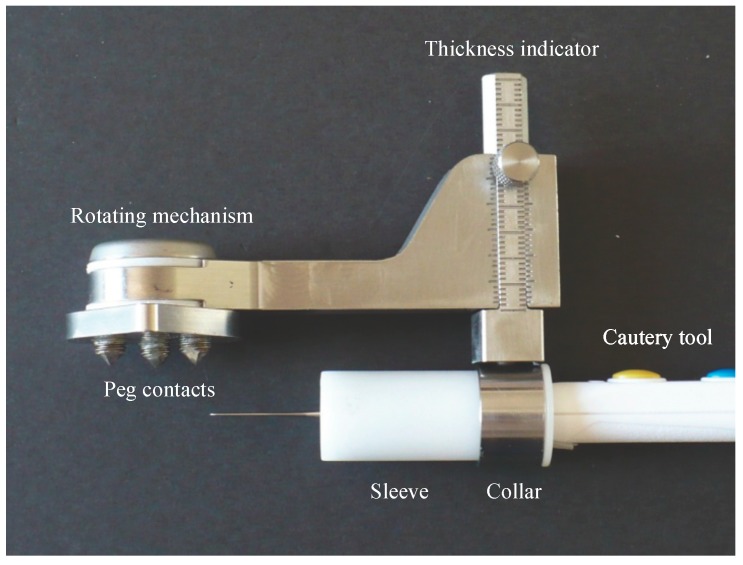
TellaMark device with cautery tool, showing the peg contacts, central rotating mechanism, and dovetail thickness indicator, suitable for all patellar shapes and sizes. The cautery tool collar is connected to the dovetail via a rare earth magnet to allow for free rotation. The cautery tool sleeve can translate freely in and out. Together, this makes it possible for the user to mark over 180° of the patella.

**Figure 2:**
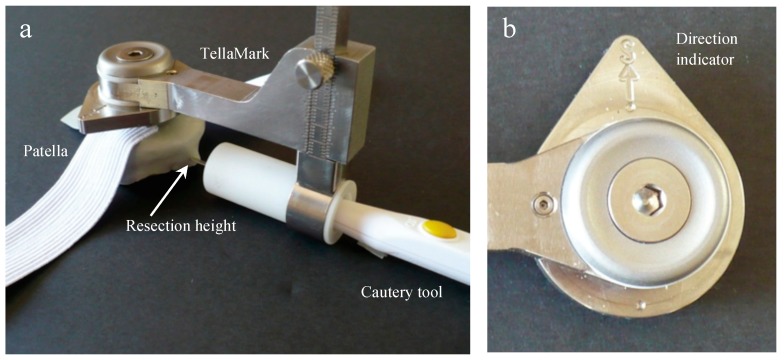
TellaMark device (**a**) placed on a patella, (**b**) with the arrow pointing superiorly. The device is placed centrally on the patella, with the pegs forming a 16 mm equilateral triangle, with two pegs positioned superiorly and one inferiorly. This configuration was determined after detailed analysis and surgeon input on 18 patellar models. The line marked by the user is then parallel to the anterior surface at the defined thickness.

The central concept of the device is to mark a line on the patella parallel to the anterior surface (i.e., around the circumference), using a cautery tool or marker pen, and then remove the device, leaving the marked line. The surgeon then uses their technique of choice (freehand, saw guide, or reamer) to align the saw or reamer with the marked line. In this way, the surgeon can continue to use the device they are most comfortable with, but with greater confidence and accuracy. By not providing a saw slot, the device is lighter, smaller and non-invasive, and leaves the control in the surgeon’s hands. The surgeon or resident can compare the drawn line to what they would have done, learning in the process, and providing a second-thought evaluation of the patellar cut. This is similar to the common practice of drawing several guidelines on the femur (transepicondylar axis, posterior condylar axis, and Whiteside’s line) to see how they compare. Residents may have the opportunity to do the patellar cut earlier in their residency because the surgeon, after checking the drawn line, will feel more confident in the resulting resection. The device is called TellaMark, to describe Marking the paTella (Figure 1 and Figure 2).

Essential to the device is accurately defining the anterior surface to achieve the desired resection. The contact configuration was determined by first having four surgeons draw lines on 18 axial and sagittal X-ray images created from computed tomography (CT) images of patellae, marking the estimated anterior surface and desired resection line, and then testing different contact configurations virtually on the 3D CT images to achieve the desired resection plane. The best configuration was determined to be a 16 mm equilateral triangle with two points superiorly and one inferiorly, centred on the patella (Figure 1). The teardrop shape, with ‘S’ marked for superior, can be easily rotated to suit a right or left patella (Figure 2). The prototype design used for testing had cone-point set screws as the contact points to allow their depth to be adjusted during the initial stages of testing. The length was chosen to be short enough to promote stability while being long enough to allow visibility when applying the device to the anterior surface. The size and sharpness were tested to grab onto the bone without digging in excessively.

Rotating the cautery tool around the patella is achieved using a swing arm, a strong rare earth magnetic coupling, and a custom Delrin sleeve that fits around the cautery tool, sliding in and out of the metal collar (Figure 1). This sleeve could have a different inner profile for cautery tools with a different shape. The device could also be used with a marker pen, but the surgeons and residents preferred the cautery tool as it is more reliable and leaves a finer line. By pushing the cautery tool in and out of the metal collar while rotating it around the patella, the line can be drawn more than 180° around the patella, posterior to the tendon attachments, providing guidance in both the ML and SI directions.

The desired depth is set on the sliding dovetail mechanism (Figure 1 and Figure 2), allowing the surgeon continuous depth adjustment. It was originally intended to be set exactly at the desired depth, but through this testing we discovered that it is advantageous to set the depth slightly thinner, resecting posterior to the line instead of on it, so that alignment with the marked line can be checked following resection. This could be part of the instruction procedure or could be incorporated directly into the device.

The device is held onto the patella with the thumb and forefingers, to avoid using an invasive bone screw or bulky clamping device, and to provide haptic feedback to the surgeon when applying the device to help avoid tilting the contacts off the anterior surface. Using the thumb and fingers works because the device is only used to mark the line rather than to create the saw cut, and is only held on for a short duration of time. The resulting profile provides good visibility of the patella while marking the line. The device is suitable for all patellar shapes and sizes, medial or lateral approaches, with right- or left-handed surgeons.

To our knowledge, no other device exists in which the desired cut line is drawn on the bone surface, for this or any other joint.

### 2.2. Artificial Bone Testing

To mimic the surgical setup in the artificial bone testing, and to perform pilot testing for design and use iterations before testing on valuable and limited cadaveric specimens, medium-sized right and left legs (Sawbones, Pacific Research Laboratories Inc., Vashon, WA, USA), without patellae, were set up at full extension and anchored onto a table. Custom patellae (see below) were attached to the femur and tibia models using materials simulating the tendons and lateral retinaculum, and covered with material representing skin. A standard incision represented the visibility and access during surgery.

**Figure 3:**
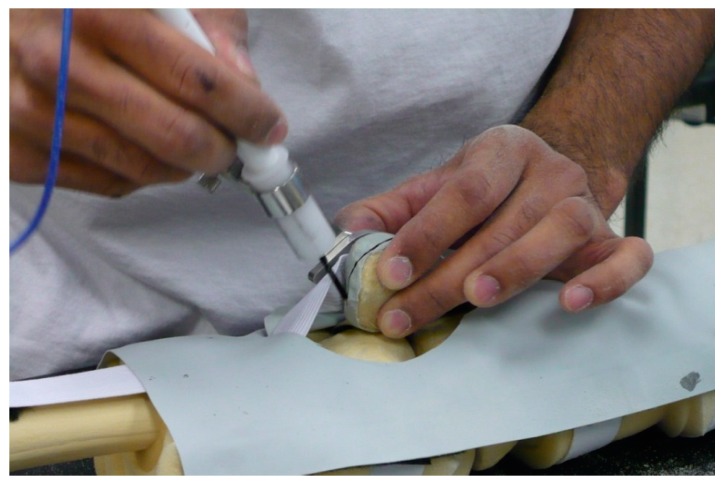
Using the TellaMark device to draw the desired resection line on artificial bones.

Two custom-molded patellar geometries were created and used in the testing phase, an approach that could be useful to other researchers, as they were more realistic than previously-used Sawbones patellae and were derived from CT scans of cadaveric specimens with which we had done earlier resection analyses and could in turn be used to compare the resulting resections in the present experiments. Geometry 1 was a left patella, smaller, regularly-shaped, and considered the ‘easier’ geometry, based on the most consistent estimated resection lines drawn by surgeons on pseudo-X-rays generated from the CT scans. Geometry 2 was a right patella, larger, irregularly-shaped, and considered the ‘harder’ geometry based on the least consistent estimated resection lines. Patellar bone models were generated from the CT scans and rapid prototyped. A mold made from the rapid-prototyped model was used to generate the patellar bone models (Foam-it 15; SmoothOn Inc., Easton, PA, USA, for which the density is 15 pounds per cubic foot). The anterior surface was covered with a thin layer of Thera-band to provide compliance and to partially obscure the anterior surface. Since the foam is insulating and the cautery tool requires a conduction path, the experimenters instead dipped the cautery tool in calligraphy ink, leaving an ink line on the patella (Figure 3). Normal use of the cautery tool was verified during the cadaveric testing.

**Figure 4:**
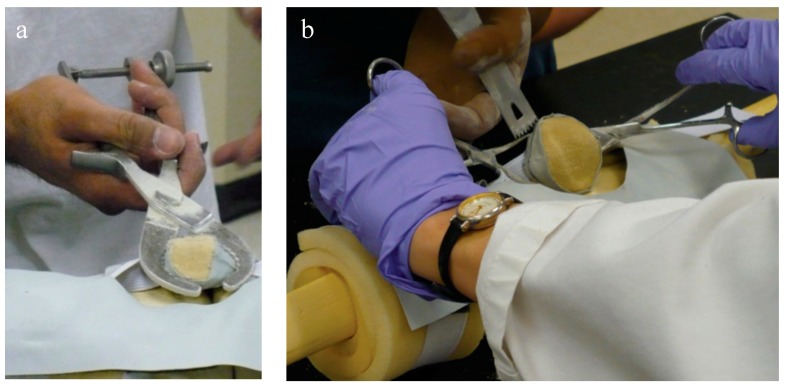
(**a**) Applying the saw guide to the patella and (**b**) securing the patella with towel clips for freehand resection. It worked best to mark the line slightly posterior to the desired thickness so that the line remained on the patellar bone remnant after resection to confirm the final cut, taking this into consideration in the thickness setting.

Two orthopaedic surgery residents (4th and 5th year) performed resections using three techniques: (1) using the conventional technique with a standard surgical saw guide (Zimmer; Warsaw, IN, USA); (2) TellaMark with the saw guide; and (3) TellaMark using a freehand technique, in each case with a surgical oscillating saw (Figure 4).

For the TellaMark resections, the initial resection was left as it was, whereas for the conventional resections, the experimenter measured the thickness and symmetry with calipers and had the option of revising the cut until satisfied. After initial practice with the instruments and experimental setup, each experimenter performed three repetitions of each of the three techniques on the two different geometries, for a total of 18 tests each. Tests were performed in a randomized order. The procedure time was recorded, including a breakdown of the steps.

The TellaMark procedure began by locating the center of the patella; this was done by feeling the height and width with the fingers, and marking the resulting central point with a marker pen or cautery tool (in the future, a dedicated device can be developed). The desired remaining thickness, determined from the patellar thickness minus the prosthesis height, was set on the depth gauge of the device and the device applied to the center of the patella with the arrow pointing superiorly. The line was then drawn with the cautery tool more than 180° around the patella, allowing both the ML and SI planes to be guided. The device was removed and the experimenter either aligned the saw or saw guide with the line to complete the cut.

The patellae were CT scanned before and after resection (0.6 mm slice thickness), followed by segmentation of the patellar bone (Amira Version 5.3.1; Visage Imaging, Andover, MA, USA). The resected patellae were aligned to the original surface models using the AlignSurface function in Amira, plus manual fine tuning, and then brought into AutoCAD (Version 2010, AutoDesk, San Rafael, CA, USA). In AutoCAD, an average plane was fitted visually to the resected surface of the patellar model, and then the average resection plane, determined previously from the four surgeons’ input on pseudo X-rays, was applied to the model. This was the particular advantage of making custom molds of the previously-analyzed patellae. The ML and SI angles were measured between the resultant plane and the average surgeon-identified resection plane. The center of the patella was determined from the medial, lateral, superior, and inferior extents of the model, i.e., by drawing a box around the patella. The thickness from the anterior surface to the resected surface was measured at this central point and then compared to the intended remaining thickness specified in the testing process (13 mm for the left, 12 mm for the right).

The angle, thickness and time data were analyzed using ANOVA, followed by Student’s *t*-tests when significant, using PASW Statistics 17.0 analysis software (Statistical Package for Social Sciences (SPSS) Inc., Chicago, IL, USA). Shapiro-Wilk tests confirmed the normality of the data. Angles within ±7° were considered symmetric based on previous studies that showed greater anterior knee pain beyond this limit and represented a normal range of results [8,10].

### 2.3. Cadaveric Testing

Eight pairs of fresh-frozen cadaveric knee specimens (six female, two male; mean age 82, range 67 to 90 years) were used for testing, following ethics approval. They were CT scanned prior to testing and then prepared with a midline incision followed by a standard parapatellar capsulotomy: medial in 14 cases, lateral in two, providing the opportunity to test both approaches. The soft tissues were released to allow for eversion of the patella, and cleared around the circumference to allow for the application of the saw guide, as done clinically. The specimens varied from no arthritis to severe arthritis, with the majority having moderate arthritis (grades 2–3). The arthritic state did not affect the experiment since the device relies only on the anterior surface, not the articulating surface, one of the advantages of the device.

**Figure 5:**
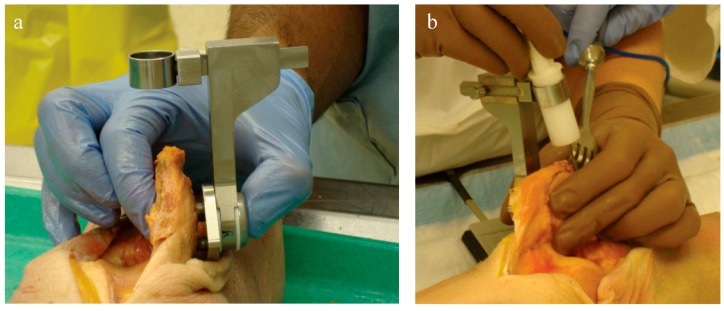
(**a**) Applying TellaMark to a cadaveric specimen; and (**b**) making a cautery line around the patellar circumference. Use of the thumb and forefingers provides haptic feedback on the contact of the device, leading to quick installation and removal. A single-pointed clamp (so as to keep the peg contacts on the surface) similar to towel-clamps could also be used to make the process hands-free.

**Figure 6:**
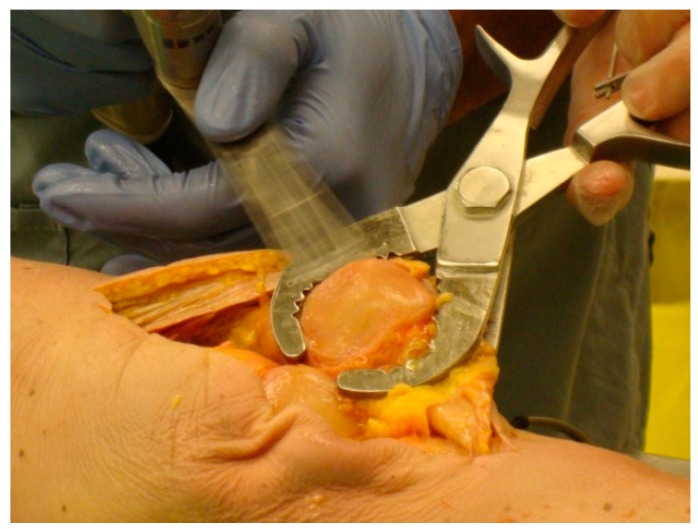
Using the conventional saw guide on a cadaveric specimen. Saw-guide use depends highly on the experience of the surgeon and the shape of the patella.

For each specimen pair, a TellaMark with saw-guide resection was performed on one side (Figure 5) and a conventional saw-guide resection was performed on the other (Figure 6), in a randomized order. The same two senior residents who performed the artificial bone testing performed the cadaveric testing. The cautery tool produced a clear, precise line, about 1 mm in thickness. As with the artificial bones, in the TellaMark case, the first cut was taken as the final cut; small corrections to the resection were allowed, such as removing a ridge, but the resection plane itself was not allowed to be recut or otherwise modified. In the conventional case, the experimenter could correct the cut until satisfied; cuts after the initial saw-guide cut were usually done freehand, with the patella being secured with towel clips (Figure 4). Experimenter 1 set the TellaMark device in such a way that the line would be cut off with the saw; Experimenter 2 set it in such a way that the saw cut just above the line, leaving the line visible afterward. This latter technique had the advantage of confirming that the cut made corresponded to the cut recommended by the device, and is now the recommended technique. The desired thickness was determined from caliper measurements, with the prosthesis thickness being subtracted from the total thickness.

Once the resections were complete, CT images were acquired and used to calculate the desired resection plane as well as the achieved resection plane for each patella, by importing the segmented surfaces into AutoCAD. From this the ML and SI angles as well as the remaining bone thickness were measured, using the same method as for the artificial bone models. The three-peg model of the device was applied to the surface to determine the expected TellaMark resection angle. ANOVA tests of the ML angle, SI angle, bone remnant thickness, and time results were performed with *p* < 0.05 being considered significant. Normality was confirmed.

## 3. Results

The TellaMark device produced comparable or better results on average when compared to the conventional saw-guide technique, with an average reduction in time. In most cases, the results were not statistically different. Almost all of the ML angles were within the symmetry limit, i.e., acceptable. The cadaveric SI angles were mostly outside the symmetry limit for the conventional technique. Individual results are discussed below.

### 3.1. ML Resection Angle

**Figure 7:**
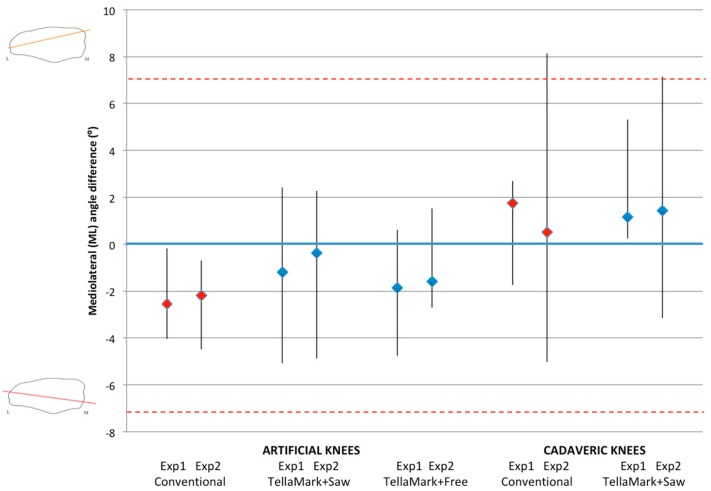
Mediolateral (ML) angle difference from the desired plane, showing the median, minimum and maximum, for the artificial bone models and cadaveric specimens, using the conventional saw guide (red markers), TellaMark + saw guide and TellaMark + freehand (blue markers). The dashed red lines indicate the symmetry goal (±7°). The one TellaMark outlier had a lateral approach and capsulotomy, which the experimenter found confusing because of the truncated specimen.

There were no significant differences in ML resection accuracy between techniques in either the artificial or cadaveric testing (Figure 7). All but two of the conventional and one of the TellaMark angles were within the symmetry thresholds (Figure 7, red dashed lines). On average, the ML angles when using TellaMark were slightly closer to the desired angle than with the conventional technique.

### 3.2. SI Resection Angle

**Figure 8:**
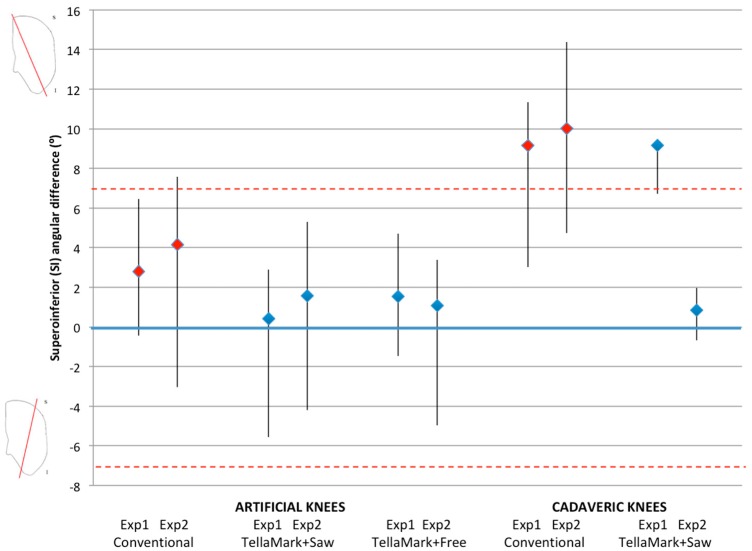
Superoinferior (SI) angle difference from the desired plane, showing the median, minimum and maximum, for the artificial bone models and cadaveric specimens, using the conventional saw guide (red markers), TellaMark + saw guide and TellaMark + freehand (blue markers). The dashed red lines indicate the symmetry goal (±7°). The substantially more accurate TellaMark vs. conventional results for Experimenter 2 vs. Experimenter 1 may be due to leaving the guideline visible (which is now recommended) and carefully centering the device on the patellar surface (a centering device will be added in the future).

For the artificial results, all but one conventional resection and all of the TellaMark results were within the symmetry limit (Figure 8, red dashed lines). By contrast, for the cadaveric results, all but two of the conventional results were outside of the symmetry limit. 

There were no significant differences in SI resection accuracy between techniques in either the artificial or cadaveric testing (Figure 8). On average, the SI angles when using TellaMark were closer to the desired angle than with the conventional technique. The TellaMark results differed by experimenter: Experimenter 1, who cut off the guide line during the resection, had similar SI accuracy to the conventional technique (median, 9.2° in each case); Experimenter 2, who retained the guide line during the resection, had dramatically better results with TellaMark (median 0.9° with TellaMark compared to 10° for the conventional technique; *p* = 0.02).

### 3.3. Bone Remnant Thickness

In the artificial bone testing, there were no significant differences in thickness between the three techniques (averaging 0.6 mm, 0.5 mm, and 0.7 mm thinner than intended, for the conventional technique, TellaMark + saw guide and TellaMark + freehand, respectively, with standard deviations of 0.6 mm, 1.0 mm, and 1.0 mm, respectively). In the cadaveric testing, the bone remnant tended to be thinner than intended with the conventional technique (−0.9 ± 0.5 mm) and thicker than intended with the TellaMark + saw guide (1.0 ± 1.0 mm), resulting in a significant difference between the two techniques (*p* < 0.001), although both averaged close to the intended thickness.

### 3.4. Procedure Time

In the artificial bone testing, there were no significant differences in time between techniques (3.8 ± 1.1 min for TellaMark + saw guide, 3.6 ± 0.7 min for TellaMark + freehand, 4.0 ± 0.7 min for the conventional technique), however the time breakdowns did differ, with more time required to perform recuts with the conventional saw guide whereas TellaMark required more initial time leading up to the resection. The main time consumption in both cases related to securing the patella with the saw guide or towel clips, and performing the cut. The time taken was significantly different between experimenters (*p* = 0.002), whereby Experimenter 2 took 50 s longer on average to complete a resection.

In the cadaveric testing, resection time was significantly shorter with TellaMark than the conventional technique (4.8 ± 1.8 min for TellaMark vs. 7.2 ± 1.2 min for the conventional technique; *p* = 0.03). As with the artificial bone testing, the TellaMark device took more time initially whereas the conventional device took more time later.

## 4. Discussion

In this study, a novel device for patellar resection was tested on artificial bone models and cadaveric specimens, demonstrating symmetries that were equivalent or better on average than the conventional technique in a similar amount of time, with a substantial improvement in SI symmetry (0.9° for TellaMark vs. 10° for the conventional technique) for the experimenter who retained the TellaMark guide line, even with multiple cuts permitted for the conventional technique, in contrast to the single cut permitted with TellaMark. Further improvements in the TellaMark design, based on this testing, together with more experience using the device, should lead to additional increases in accuracy and reductions in time.

Because of the advantages of keeping the guideline visible, we now recommend setting the depth greater by 1 mm, or building this into the device. The outlier ML cadaveric resection was likely caused by the lateral approach, which the experimenters said threw them off (the truncated knee specimen can easily be turned one way or the other, so the directions were less intuitive) as well as the hard, sclerotic bone, which the saw had trouble getting through.

For the SI resections, Experimenter 1 appears to have mounted the device higher than the patellar center (based on later analysis of the CT scans), which caused the device to have an angle relative to the anterior surface. In the future, we recommend incorporating a viewing hole that can be placed over the central mark to ensure that the desired centering is achieved, as well as possibly adding a device or procedure to aid with the centering. Experimenter 2 took on average 30 s longer than Experimenter 1 to ensure that the device was centred. A simple mechanical device could achieve this centering quickly and without subjectivity. The apparent value for SI symmetry is important since a previous study showed that SI symmetry was even more strongly correlated to anterior knee pain (*p* = 0.001) than ML symmetry (*p* = 0.02) [4]. 

Regarding thickness, TellaMark can be used to resect at the final depth directly, or can be used first with a conservative thickness to guide the angle, followed by a final cut after checking the thickness. This could be particularly valuable if the patella is not everted. If there is a consistent bias, e.g., to a thicker patellar as in this study, this can be incorporated into the design and instructions of the device.

A related device that uses the same three-peg contact on the anterior surface, designed to verify the patellar cut after it is made, demonstrated good accuracy [12], suggesting that deviations in this study related mainly to the execution of the cut and that the estimation of the anterior surface is robust.

The main limitation of this study was the small number of experimenters and specimens, reflecting the availability of specimens and the time required to perform the testing. Also, while the artificial bone testing provided useful feedback on the design and use of the device as well as practice for the experimenters, it did not fully mimic the cadaveric testing. A larger cadaveric study should be conducted once the design is updated based on the results of this testing; nonetheless, the present study was able to reveal the fundamental similarities and differences between the TellaMark and conventional results. One other consideration is that conventional techniques are relatively successful in a lab setting (i.e., within the symmetry limit), despite wide ranges clinically, making it difficult to show a clear advantage of a new device, especially given the multiple cuts allowed for the conventional technique but not for the new device. After further testing to ensure the best usability and accuracy of TellaMark, our goal is to implement the device clinically, allowing expert surgeons to compare their intended resection with the proposed resection and then examine the final resection result to determine which would have been the better choice. 

For clinical use, there should be fewer parts, which can be addressed through higher-volume manufacturing methods, and the plastic bearing should be replaced by metal for ease of sterilization. While the thumb and forefingers provide a good way of holding the device on the patella, it is also possible to hold it in this position mechanically: a pointed connection or small contact point similar to a towel clip can help hold it in place to release the fingers for applying the cautery mark; a clamp with a large contact area should be avoided as this tends to tilt the contact points off the anterior surface or tilt the patella [8]. To our knowledge, the concept of marking bone to indicate the desired resection plane has not been used previously and could benefit other joints as well.

The experimenters considered the device a useful learning tool to create better resections, especially for less experienced surgeons. They appreciated the guidance that it provided, so that they did not need to struggle to decide where to put the saw guide. They also appreciated the fact that the resident or surgeon could confirm that they are satisfied with the line before proceeding with the cut. The expert surgeons we interviewed like that it provides guidance without constraining them to a particular resection line, and allows them to continue using the freehand or saw guide technique that they are already familiar with.

## 5. Conclusions

With minor design and use changes to address the current outliers, TellaMark offers the potential to substantially improve patellar resection thickness and symmetry, particularly given the substantially better SI results for Experimenter 2 compared to the conventional technique. It also offers the opportunity to improve training and confidence. On the basis of previous studies, better patellar resection accuracy will translate to reduced pain and increased function after knee replacement surgery, leading to more satisfied patients.
